# Prevalence and molecular characterization of *Giardia intestinalis* isolated from children and calves in Babylon province, Iraq

**DOI:** 10.14202/vetworld.2023.1781-1789

**Published:** 2023-09-13

**Authors:** Haider H. Alseady, Sahad M. K. Al-Dabbagh, Ali D. Marhash

**Affiliations:** 1 Technical Institute of Babylon, Al-Furat Al-Awsat Technical University, 51015, Babylon, Iraq; 2 Institute of Medical Technology Al-Mansour, Middle Technical University, 10001, Baghdad, Iraq

**Keywords:** assemblages, calves, children, *Giardia intestinalis*, nested polymerase chain reaction, triose phosphate isomerase, prevalence

## Abstract

**Background and Aim:**

*Giardia intestinalis* is one of the most prevalent intestinal parasites in humans and animals, and children in close contact with livestock are particularly at risk of infection. This study aimed to detect assemblages of *G. intestinalis* and determine the origin of zoonotic transmission of *Giardia* in children and calves in different parts of Babylon province, Iraq.

**Materials and Methods:**

One hundred stool samples from children (68 boys and 32 girls) and 100 fecal samples from calves (46 males and 54 females) of different ages were randomly collected. Molecular techniques were used to estimate the prevalence of *G. intestinalis* in children and calves. A nested polymerase chain reaction (PCR) was performed by targeting the triose phosphate isomerase gene in the samples to detect *G. intestinalis* assemblages.

**Results:**

The overall rates of infection with *G. intestinalis* in children and calves were 21% and 34%, respectively, using the conventional microscopic method. The results illustrated that 61.90% (13/21) and 38.09% (8/21) of positive samples from children were allocated to assemblages A and B, respectively (p > 0.05). In calves, assemblages A and B were detecte in 82.35% (28/34) and 17.64% (6/34) of positive samples from calves, respectively (p ≤ 0.001). Ten PCR products were sequenced and submitted to the GenBank database. Phylogenetic analysis detected five human sequences each belonging to *G. intestinalis* assemblages A (OM850335–OM850339) and B (OM850340–OM850344). Similarly, five calf sequences each belonged to *G. intestinalis* assemblages A (ON75756–ON757660) and B (ON757661–ON757665).

**Conclusion:**

The detection of large numbers of *G. intestinalis* assemblage A in both humans and cattle indicated that cattle could be a main source of zoonotic *G. intestinalis* infection in children in Babylon province, Iraq.

## Introduction

*Giardia intestinalis* (syn. *Giardia duodenalis and Giardia lamblia*) is an intestinal flagellated protozoan responsible for acute and chronic diarrhea, with most infections being asymptomatic [1]. Chronic infection is associated with malabsorption, which leads to weight loss and wasting in children, resulting in decreased quality of life [2, 3]. *Giardia intestinalis* is considered one of the most important zoonotic protozoan parasites affecting humans and a wide range of domestic animals globally [4, 5]. The World Health Organization reported more than 200 million symptomatic infections caused by *G. intestinalis* in developing countries of Africa, Asia, and Latin America [6] and approximately 280 million annual cases worldwide [7]. In contrast, the prevalence of *Giardia* infection in cattle varies by area, ranging from 9% to 73% [8]. Eight genetic assemblages of *G. intestinalis* (A–H) have been detected. The major assemblages of potential zoonotic importance found in humans and animals (A and B) are further divided into eight sub-assemblages that exhibit preferences for human and/or animal hosts (AI, AII, AIII, AIV, BI, BII, BIII, and BIV) [9, 10].

Different transmission pathways, including the fecal–oral route through the ingestion of cysts, person-to-person transmission, and zoonotic transmission, have been described [11–13]. Food and water contaminated with cysts have been reported to have an infectious dose as small as ten cysts, leading to *Giardia* infection outbreaks [14].

Microscopic detection of *Giardia* cysts in fecal samples from different hosts is the most frequent detection method. Despite being a simple technique that provides valuable information, microscopic detection is labor-intensive, it requires substantial time, it lacks sensitivity, and depends on the microscopic skills of the operator. Serological diagnosis, including enzyme-linked immunosorbent assay, had been developed to detect *Giardia* antigens in stool. However, problems with false–positive and false–negative test results have been reported [15].

Polymerase chain reaction (PCR) has greatly improved the detection of *Giardia* and provided epidemiological data with higher sensitivity and specificity than microscopic and serological methods. These techniques use the triose phosphate isomerase (tpi) gene for the molecular detection of *G. intestinalis* due to its high genetic heterogeneity and polymorphism [16].

This study aimed to determine the origin of *G. intestinalis* infection and molecularly characterize *G. intestinalis* assemblages in children and calves in Babylon province in Iraq.

## Materials and Methods

### Ethical approval

The Animals and Ethics Committee of Al-Furat Al-Awsat University approved (BMS/0231/016) the study to collect samples from calves, the samples collected from children in different regions of Babylon Province, Iraq.

### Study period and location

This study was conducted from September 5, 2021, to June 1, 2022, in the Laboratory of Medical Laboratory Techniques, Technical Institute of Babylon, Al-Furat Al-Awsat University.

### Samples collection

One hundred stool samples (68 male and 32 female) were randomly collected from children of different ages (1–12 years) in rural areas of Babylon province, Iraq, who were in contact with cattle, and 100 fecal samples (46 male and 54 female) were collected from calves (1–12 months old) in different parts of the same province. In total, 15–20 g of fecal samples were taken from each individual.

### DNA extraction

DNA extraction was performed using a fecal lysis protocol with proteinase K according to the manufacturer’s instructions (Stool DNA extraction kit, Bioneer, Korea). Then, the extracted gDNA was analyzed using a NanoDrop spectrophotometer (Thermo Fisher, USA) and stored at −20°C in a refrigerator until use for PCR amplification [17].

### Molecular technique

The nested PCR technique was used to detect *G. intestinalis* assemblages A and B in stool samples from children and calves as described previously by Minvielle *et al*. [18]. Primers targeting the tpi gene (Table-1) were acquired from Bioneer.

**Table-1:** The nested PCR primers used for the detection of *Giardia intestinalis*.

Nested PCR	Primer	Sequence		Amplicon
First round	Tpi A	F	5’-CGAGACAAGTGTTGAGATG-3’	576 bp
		R	5’-GGTCAAGAGCTTACAACACG-3’	
	Tpi B	F	5’-GTTGCTCCCTCCTTTGTGC-3’	208 bp
		R	5’-CTCTGCTCATTGGTCTCGC-3’	
Second round	nTpi A	F	5’-CCAAGAAGGCTAAGCGTGC-3’	535 bp
		R	5’-GGTCAAGAGCTTACAACACG-3’	
	nTpi B	F	5’-GCACAGAACGTGTATCTGG-3’	470 bp
		R	5’-CTCTGCTCATTGGTCTCGC-3’	

PCR=Polymerase chain reaction, tpi=Triose phosphate isomerase

The PCR premix tube contained a freeze-dried pellet of 1 U of Taq DNA polymerase, 250 μM deoxynucleoside triphosphates, 10 mM Tris-HCl (pH 9.0), 1.5 mM MgCl_2_, 30 mM KCl 30, stabilizer, and tracking dye. The PCR master mix was prepared according to the kit instructions in a total volume of 20 μL by adding 5 μL of purified genomic DNA and 1.5 μL each of forward and reverse primers (10 pmol). Deionized water was added to increase the volume to 20 μL, and the mixture was mixed using an ExiSpin vortex centrifuger (Cyan, Belgium). The reaction conditions were initial denaturation at 95°C for 5 min; 30 cycles of denaturation at 95°C for 30 s, annealing at 52°C for 30 s, and extension at 72°C for 1 min; and final extension step at 72°C for 7 min. The PCR products were examined by electrophoresis (Shandod Scientific, UK) in a 1.5% agarose gel, stained with ethidium bromide, and visualized under an ultra-violet transilluminator (Atta, Korea).

### Sequence analysis

Genetic analysis was performed by phylogenetic tree analysis between local *Giardia* spp. isolates from children and calves and *Giardia* spp. submitted to National Center for Biotechnology Information Blast. The identified isolates were then submitted to GenBank. Ten positive PCR small subunit ribosomal RNA gene products from each sample were shipped to Macrogen Company, Korea, on ice for DNA sequencing using the AB DNA sequencing system (Macrogen Company). DNA sequencing analysis was performed using Molecular Evolutionary Genetics Analysis version 6.0 ( https://www.megasoftware.net/), and multiple sequence alignment analysis of the partial small subunit rRNA gene using ClustalW ( http://www.clustal.org). and calculation of the evolutionary distances were performed by the maximum composite likelihood method using the unweighted pair group method with arithmetic mean method [19].

### Statistical analysis

Statistical analyses were performed using the statistical package for the social sciences version 17 (IBM Corp., NY, USA). Significant differences in variables were determined using the Chi-squared test. Highly significant differences were indicated by p ≤ 0.001, and a lower significant differences were indicated by p ≤ 0.05 [20].

## Results

### Prevalence of *Giardia*

Conventional microscopic analysis revealed that the overall prevalence rates of *Giardia* were 21% in children and 34% in calves (Table-2).

**Table-2:** Prevalence of *Giardia* infection using conventional microscopic method in children and calves.

Host	No. of samples examined	No. of positive	Infection rate %
Children	100	21	21
Calves	100	34	34

**Figure-1:**
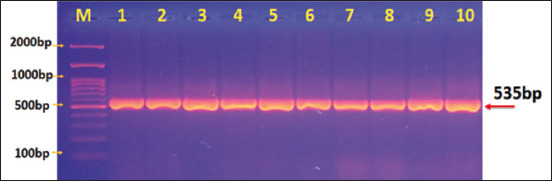
Agarose gel electrophoresis image that showed polymerase chain reaction (PCR) product analysis of TPI gene in *Giardia intestinalis* assemblage A of stool samples. (M) Marker ladder (2000–100 bp). Lane (1–10) *G. intestinalis* assemblage A at 535 bp PCR product.

**Figure-2:**
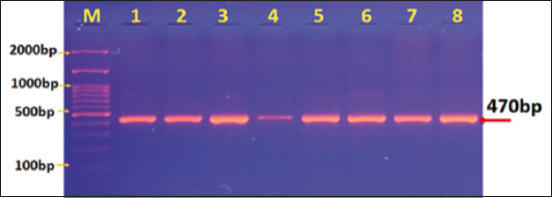
Agarose gel electrophoresis image that showed polymerase chain reaction (PCR) product analysis of triose phosphate isomerase gene in *Giardia intestinalis* assemblage B of stool samples. (M) Marker ladder (2000–100 bp). Lane (1–8) *G. intestinalis* assemblage B at 470 bp PCR product.

The amplified gene products obtained from stool samples from children and calves were subjected to nested PCR using primers targeting the tpi gene to identify assemblages of *G. intestinalis*. The results were confirmed by agarose gel electrophoresis. A distinct band of 535 bp was identified for *G. intestinalis* assemblage A (Figure-1), and a 470-band was observed for *G. intestinalis* assemblage B (Figure-2).

### Rate of infection by different *G. intestinalis* assemblages

Children were more frequently infected by assemblage A (13/21, 61.90%) than by assemblage B (8/21, 38.09%), although without significance. In calves, the rates of infection by assemblages A and B were 82.35% (28/34) and 17.64% (6/34), respectively (p ≤ 0.001, Table-3).

**Table-3:** Rate of infection with *G. duodenalis* according to assemblage.

*G. duodenalis* assemblage	No. of sample examined	No. of positive	Infection rate %	p-value
Children				
Assemblage A	21	13	61.90	p ≤ 0.05
Assemblage B	21	8	38.09	
Calves				
Assemblage A	34	28	82.35	p ≤ 0.001
Assemblage B	34	6	17.64	

*G. duodenalis=Giardia duodenalis*

### *Giardia intestinalis* genotypes in children and calves

Ten samples were positive for *Giardia* by nested PCR amplification of the tpi gene, and they were successfully sequenced. The genotyping results revealed the presence of two *Giardia* genotypes (A and B), and both sequences were 99% identical in humans and cattle (Tables-4 and 5).

**Table-4:** NCBI-BLAST Homology sequence identity (%) between local *G. intestinalis* human isolates and NCBI-BLAST submitted *G. intestinalis* assemblage isolates.

*Giardia* genotype isolates	GenBank accession No.	NCBI-BLAST homology sequence identity		

		Identical *Giardia*	GenBank accession No.	Identity (%)
IQ-Human No. 1	OM850335.1	Assemblage A	LC329330.1	99.87
IQ-Human No. 2	OM850336.1	Assemblage A	LC329330.1	99.62
IQ-Human No. 3	OM850337.1	Assemblage A	LC329330.1	99.14
IQ-Human No. 4	OM850338.1	Assemblage A	LC329330.1	99.13
IQ-Human No. 5	OM850339.1	Assemblage A	LC329330.1	99.78
IQ-Human No. 6	OM850340.1	Assemblage B	KY444789.1	99.17
IQ-Human No. 7	OM850341.1	Assemblage B	KY444789.1	99.19
IQ-Human No. 8	OM850342.1	Assemblage B	KY444789.1	99.13
IQ-Human No. 9	OM850343.1	Assemblage B	KY444789.1	99.76
IQ-Human No. 10	OM850344.1	Assemblage B	KY444789.1	99.15

NCBI-BLAST=National Center for Biotechnology Information-Basic Local Alignment Search Tool, *G. intestinalis=Giardia intestinalis*, IQ=Intelligence quotient

**Table-5:** NCBI-BLAST homology sequence identity (%) between local *G. intestinalis* Cattle isolates and NCBI-BLAST submitted *G. intestinalis* assemblage isolates.

*Giardia* genotype isolates	GenBank accession No.	NCBI-BLAST Homology sequence identity		

		Identical NCBI genotype	GenBank accession No.	Identity (%)
IQ-Cattle No. 1	ON75756.1	Assemblage A	LC329330.1	99.67
IQ-Cattle No. 2	ON75757.1	Assemblage A	LC329330.1	99.34
IQ-Cattle No. 3	ON75758.1	Assemblage A	LC329330.1	99.17
IQ-Cattle No. 4	ON75759.1	Assemblage A	LC329330.1	99.18
IQ-Cattle No. 5	ON75760.1	Assemblage A	LC329330.1	99.34
IQ-Cattle No. 6	ON75761.1	Assemblage B	KY444789.1	99.54
IQ-Cattle No. 7	ON75762.1	Assemblage B	KY444789.1	99.18
IQ-Cattle No. 8	ON75763.1	Assemblage B	KY444789.1	99.16
IQ-Cattle No. 9	ON75764.1	Assemblage B	KY444789.1	99.67
IQ-Cattle No. 10	ON75765.1	Assemblage B	KY444789.1	99.55

NCBI-BLAST=National Center for Biotechnology Information-Basic Local Alignment Search Tool, *G. intestinalis=Giardia intestinalis*, IQ=Intelligence quotient

### Phylogenetic analysis

**Figure-3:**
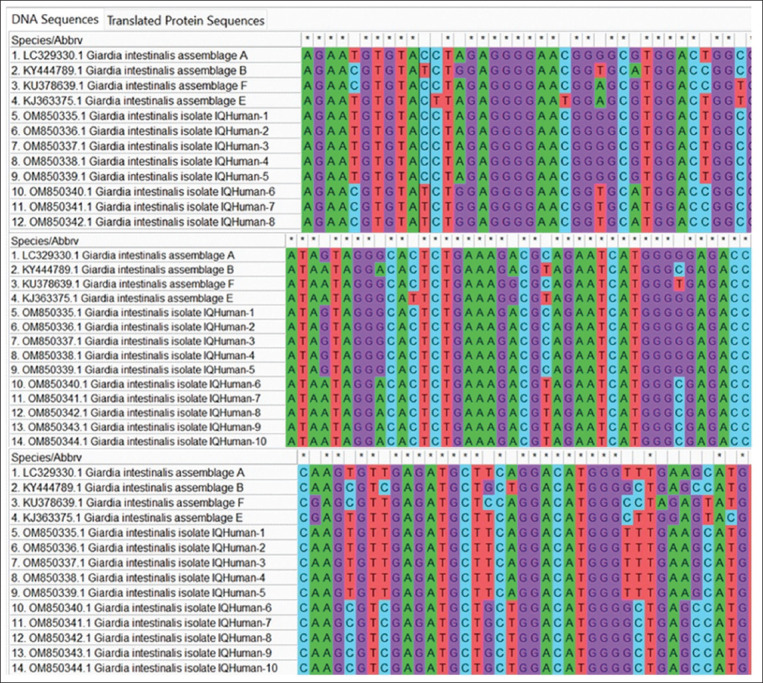
Multiple sequence alignment analysis of on triose phosphate isomerase (tpi) gene in local *Giardia intestinalis* IQ-Human isolates and National Center for Biotechnology Information-Genbank *G. intestinalis* genotypes isolates. The multiple alignment analysis was constructed using the Clustal W alignment tool in (Molecular Evolutionary Genetics Analysis 6.0 version). This showed nucleotide alignment similarity as (*) and substitution mutations on tpi gene.

**Figure-4:**
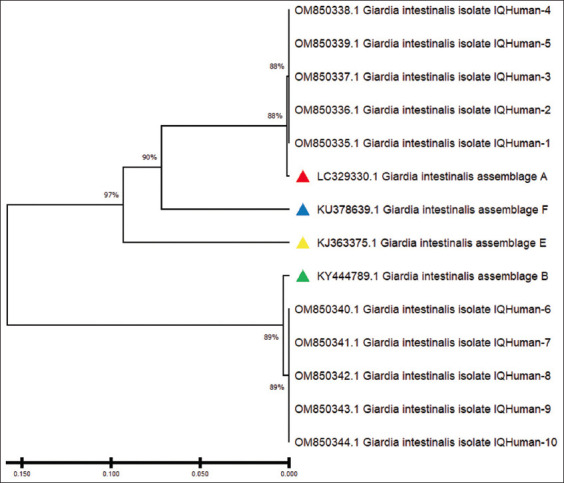
Phylogenetic tree analysis based on triose phosphate isomerase gene partial sequence in local *Giardia intestinalis* intelligence quotient (IQ)-Human isolates that are used for genetic relationship analysis. The phylogenetic tree was constructed using the unweighted pair group method with arithmetic mean (UPGMA tree) in (Molecular Evolutionary Genetics Analysis 6.0 version). The local *Giardia intestinalis* IQ.No.1 Human – IQ.No.1-IQ.NO.5 isolate were showed closed related to National Center for Biotechnology Information-Basic Local Alignment Search Tool (NCBI-BLAST) *Giardia intestinalis* isolate A: Assemblage A (LC329330.1) and the local *Giardia intestinalis* IQ.No.6-IQ-No.10 Human – IQ.No.2 isolate were showed closed related to NCBI-BLAST *Giardia intestinalis* isolate A: Assemblage B (KY444789.1) at total genetic changes (0.0150%–0.050%).

**Figure-5:**
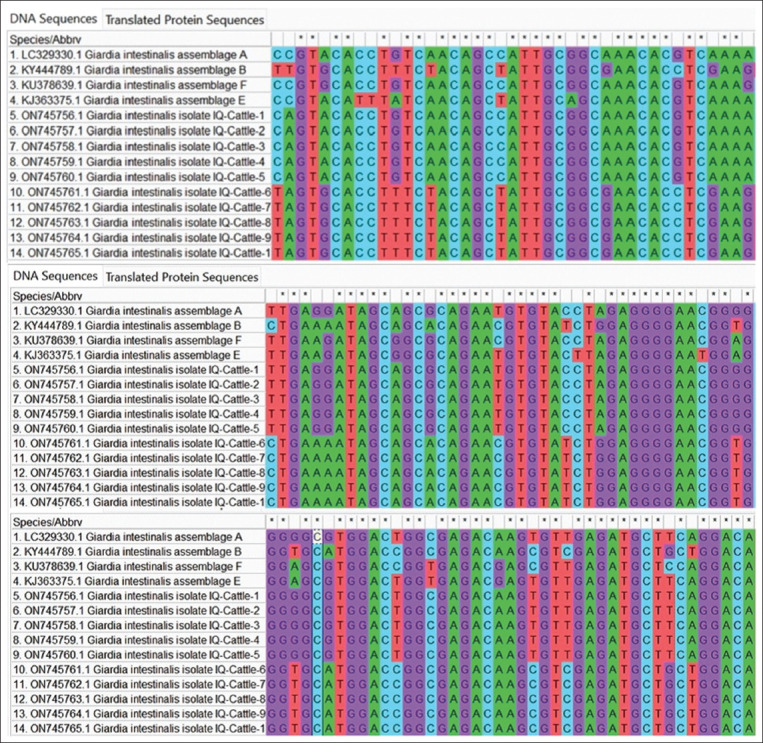
Multiple sequence alignment analysis of on triose phosphate isomerase (tpi) gene in local *Giardia intestinalis* intelligence quotient-Cattle isolates and National Center for Biotechnology Information-Genbank *G. intestinalis* genotypes isolates. The multiple alignment analysis was constructed using ClustalW alignment tool (Molecular Evolutionary Genetics Analysis 6.0 version). This showed nucleotide alignment similarity as (*) and substitution mutations on tpi gene.

**Figure-6:**
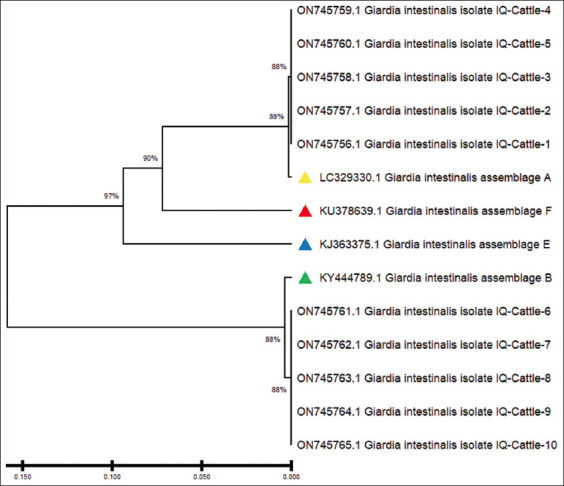
Phylogenetic tree analysis based on triose phosphate isomerase gene partial sequence in local *Giardia intestinalis* intelligence quotient (IQ)-Cattle isolates used for genetic relationship analysis. The phylogenetic tree was constructed using the unweighted pair group method with arithmetic mean (UPGMA tree) in (Molecular Evolutionary Genetics Analysis 6.0 version). The local *G. intestinalis* IQ.No.1 Cattle - IQ.No.1–IQ.NO.5 isolate was shown to be closely related to National Center for Biotechnology Information-Basic Local Alignment Search Tool (NCBI-BLAST) *G. intestinalis* isolate A: assemblage A (LC329330.1) and the local *G. intestinalis* IQ.No.6–IQ-No.10 Cattle – IQ.No.2 isolates were closely related to NCBI-BLAST *G. intestinalis* isolate A: assemblage B (KY444789.1) at total genetic changes (0.0150%–0.050%).

Ten isolates of *G. intestinalis* each were submitted to GenBank for humans (accession nos. OM850335–OM850335 for assemblage A and OM850340–OM850344 for assemblage B) and cattle (accession nos. ON75756–ON75760 for assemblage A and ON75761–ON75765 for assemblage B). The phylogenetic tree revealed the identity between the isolates of *G. intestinalis* assemblage A in both children and cattle. The isolates were similar to a *G. intestinalis* assemblage A isolate in Iran (accession No. LC329330) and a *G. intestinalis* assemblage B isolate in Iran (accession no. KY444789), as highlighted in Figures-3-6.

## Discussion

These results were in agreement with those recorded in Tikrit city, Iraq (20.7% in children aged 6–7 years) [21], Kirkuk city, Iraq (7.53%) [22], Colombia (9.9%) [23], Zambia (10%) [24], and Duhok, Iraq (5.16%) [25]. However, the observed prevalence was lower than those recorded in Al-Qadisiyah province, Iraq (54%) [26], Syria (62.5% in children aged 1–10 years) [27], Jordan (42% in children) [28], Dhi Qar province, Iraq (47.5% in children aged 1–10 years) [29], and Baghdad province, Iraq (28.5% in children) [30]. These differences could be attributable to differences in diagnostic techniques, the absence of water treatment systems and adequate sanitation, indigence, and poor hygiene, especially in rural areas.

Among 21 stool isolates from children, the prevalence was higher for *G. intestinalis* assemblage A (61.90%) than for assemblage B (38.09%). These results are in agreement with those of studies conducted in Syria (67.5% A, 10% B) [27], Egypt (75.5% A, 19.5% B) [31], Thailand (71.4% A, 2.3% B) [32], Saudi Arabia (57.5% A, 37.5% B) [33], Yemen (66% A, 34% B) [34], Ghana (60% A, 40% B) [35], and Egypt (31.4% A, 22.8% B) [36]. However, several studies conducted in Australasia [9], Zambia [24], Nepal [37], and Iraq [38] indicated that assemblage B was more prevalent than assemblage A. In this study, we used the tpi gene for the molecular detection of *G. intestinalis* due to its high genetic heterogeneity and polymorphism [39].

The diversity of *G. intestinalis* genotypes could be related to social and epidemiological criteria, different modes of transmission, and the selection of specific genes for genotype detection. It is known that in cattle, which are frequently responsible for zoonotic transmission with various animals serving as reservoir hosts, *G. intestinalis* assemblage A was the most commonly zoonotically transmitted genotype.

In calves, the observed prevalence was similar to those recorded in Basra province, Iraq (34%) [40], Babylon province, Iraq (35.5%) [41], and the United States (33.5% in weaned calves) [42]. Conversely, the observed prevalence was higher than that recorded in Baghdad province, Iraq (30%([30]. Our findings were lower than those recorded in Al-Qadisiyah province, Iraq (70%) [26], Babylon province, Iraq (47% in calves aged 1–12 months) [43], and Mosul city (50% in calves) [44]. The differences in the rates of *G. intestinalis* infection could be attributable to differences in the number of samples collected, environmental and seasonal conditions, laboratory diagnostic methods, and the ages of the animals.

In this study, zoonotic genotype A was detected in 82.35% of the positive samples of calves (28/34). Our result was in agreement with a prior study by Giangaspero *et al*. [45] that recorded rates of 42.2% and 11.1% for genotypes A and B, respectively. Other previous studies by Alhayali *et al*. [30], Ahmad *et al*. [36], Malekifard and Ahmadpour [46], and Hublin *et al*. [47] indicated that the cattle are potential reservoirs of zoonotic *G. intestinalis* in their countries.

By contrast, prior research by Al-Difaie [26], Madlol *et al*. [43] indicated that assemblage B was more prevalent than assemblage A in Iraq. This difference could be attributable to different modes of transmission, including foodborne, waterborne, and zoonotic transmission from humans to animals; the number of samples collected; the age of the animals, and the selection of genes for diagnosis.

The phylogenetic tree congregations of nitrogen bases for *G. intestinalis* assemblage A with globally registered samples have been described. The human and cattle samples in our study were asymptotic to those with the serial nos. AY228628.1 registered in Colombia [23], LC430552.1 registered in Zambia [24], LC329330 registered in Iran [48], and AB195223.1 registered in Japan [49]. The *G. intestinalis* assemblage B human and cattle samples in the present study were asymptotic to those with the serial nos. KF843922.1 registered in Colombia [23], LC430549.1 registered in Zambia [24], AY228628.1 registered in Japan [49], and KY444789 registered in Iran [50]. This dimension of the phylogenetic analysis refers to the difference in the nitrogen base successions between the local sample of humans and cattle and those registered globally.

Several studies have described the relationships between assemblages and symptoms, but no clear association was demonstrated between the *Giardia* assemblage and clinical signs. Our results illustrated no significant association between *Giardia* assemblages and symptoms, in agreement with previous findings in Syria and Iran [27, 50, 51], whereas other studies found that assemblage A was considerably associated with symptoms in Egypt [31] and Iran [52]. Meanwhile, a study in Saudi Arabia revealed that clinical symptoms were strongly related with assemblage B [53].

## Conclusion

This study described the detection of large counts of *G. intestinalis* assemblage A in both humans and cattle, indicating that cattle could be a primary source of zoonotic *G. intestinalis* infection in children in Babylon province, Iraq, and this assemblage was the most common genotype transmitted zoonotically. Control programs are suggested to reduce the risk of human infection and dangers to public health.

## Authors’ Contributions

HHA: Sample and data collection. HHA, SMKA, and ADM: Study design and drafted the manuscript and laboratory work, data analysis, and drafted the manuscript. All authors have read, reviewed, and approved the final manuscript.
